# Limited Stability of Microcystins in Oligopeptide Compositions of *Microcystis aeruginosa* (Cyanobacteria): Implications in the Definition of Chemotypes

**DOI:** 10.3390/toxins5061089

**Published:** 2013-06-06

**Authors:** Ramsy Agha, Samuel Cirés, Lars Wörmer, Antonio Quesada

**Affiliations:** Departamento de Biología, Universidad Autónoma de Madrid, C/Darwin, 2, Madrid 28049, Spain; E-Mails: ramsyagha@gmail.com (R.A.); samuel.cires@uam.es (S.C.); lars_wn@yahoo.com (L.W.)

**Keywords:** MALDI-TOF MS, chemotype, stability, nutrient deficiency, light, oligopeptide

## Abstract

The occurrence of diverse oligopeptides in cyanobacteria, including the cyanotoxins microcystins, has been recently used to classify individual clones into sub-specific oligopeptide chemotypes, whose composition and dynamics modulate microcystin concentrations in cyanobacterial blooms. Cyanobacterial chemotyping allows the study of the ecology of chemotypical subpopulations, which have been shown to possess dissimilar ecological traits. However, the stability of chemotypes under changing abiotic conditions is usually assumed and has not been assessed in detail. We monitored oligopeptide patterns of three strains of *Microcystis aeruginosa* under different nutrient and light conditions. MALDI-TOF MS revealed alterations in the microcystins signatures under N and P poor conditions and high light intensities (150 and 400 μmol photons m^−2^s^−1^). Variations in the general oligopeptide composition were caused by a gradual disappearance of microcystins with low relative intensity signals from the fingerprint. The extent of such variations seems to be closely related to physiological stress caused by treatments. Under identical clonal compositions, alterations in the oligopeptide fingerprint may be misinterpreted as apparent shifts in chemotype succession. We discuss the nature of such variations, as well as the consequent implications in the use of cyanobacterial chemotyping in studies at the subpopulation level and propose new guidance for the definition of chemotypes as a consistent subpopulation marker.

## 1. Introduction

Freshwater cyanobacteria are well known for their occasional massive proliferation in temperate water bodies during late summer periods. Cyanobacterial blooms are of public health concern, due to the ability of many genera to produce a wide range of toxins and other bioactive compounds [1]. An important group of these compounds are non-ribosomal peptides, termed onwards oligopeptides. Microcystins (MCs), a group of potent hepatotoxins, are one of these compounds.

The traditional cyanobacterial taxonomy following botanical criteria is based mainly on morphological features. However, the existence of transitional populations with wide morphological variations [2], together with the observed loss of original morphological features under culture conditions [3], implies a remarkable polymorphism, which has raised discussions about the validity of taxonomic classification with morphological criteria alone. Indeed, from a toxicological standpoint, it is well known that morphological characteristics do not correlate well with toxin profiles, which have been observed to vary widely [4].

Oligopeptides are produced by non-ribosomal peptide synthases (NRPS) [5], with the exception of cyclamides and microviridins, which are synthesized by ribosomic pathways. More than 600 structural variants of oligopeptides have been described, which have been classified in seven classes: aeruginosins, cyanopeptolins, microcystins, microviridins, microginins, anabaenopeptins and cyclamides [6]. The ability of cyanobacteria to produce oligopeptides is determined by the presence or absence of NRPS-encoding gene-clusters [7]. Cyanobacterial cells can present any combination of gene clusters, generating a specific pattern of oligopeptide production, which can be used as a characterizing oligopeptide fingerprint. The coexistence of individuals with distinct oligopeptide compositions within a population has been reported [8]. This finding allowed the use of distinct oligopeptide fingerprints as subspecific cyanobacterial markers to divide populations into oligopeptide-based subpopulations or chemotypes [9], which have been observed to possess different ecological traits [10]. Besides, MC concentrations in the course of the bloom often display marked temporal variations of up to several orders of magnitude (e.g., [11]) that represent one of the main hindrances to evaluate the risks associated with cyanobacterial blooms. Since MC is produced constitutively, its synthesis varies only about three-fold and is related to growth or cell division rates [12]. These variations cannot be explained by physiological changes at the individual level. Conversely, the relative abundance of dissimilar chemotypes in natural communities, as well as the shifts in chemotypical subpopulations, have been shown to be responsible for the observed varying MC concentrations [13] and, consequently, have to be considered critical factors modulating bloom toxicity.

Although changes in the synthesis of individual oligopeptides under changing abiotic conditions have been examined [14,15,16], these differences have not been assimilated in the context of the conservation of oligopeptide fingerprints and its evaluations as a consistent chemotype marker. Understanding potential variations in the peptide composition of clonal strains is crucial for tracking the succession of chemotypical subpopulations in natural systems and, thereby, investigate their ecology. To evaluate their stability, we tracked oligopeptide compositions of three clonal strains of *Microcystis aeruginosa* producing a wide range of microcystin variants and cultured under different scenarios. We focused on *M. aeruginosa*, a species that has been pointed out as the most important microcystin producer in Mediterranean latitudes [11,17]. Selected strains were exposed to N and P reductions and different light intensities. Their oligopeptide contents were analyzed by matrix assisted laser desorption/ionization time of flight mass spectrometry (MALDI-TOF MS) to determine whether physiological stress causes alterations in oligopeptide fingerprints, which may have major implications when delimiting chemotypes and interpreting their dynamics under natural conditions.

## 2. Results

### 2.1. Oligopeptide Compositions

**Figure 1:**
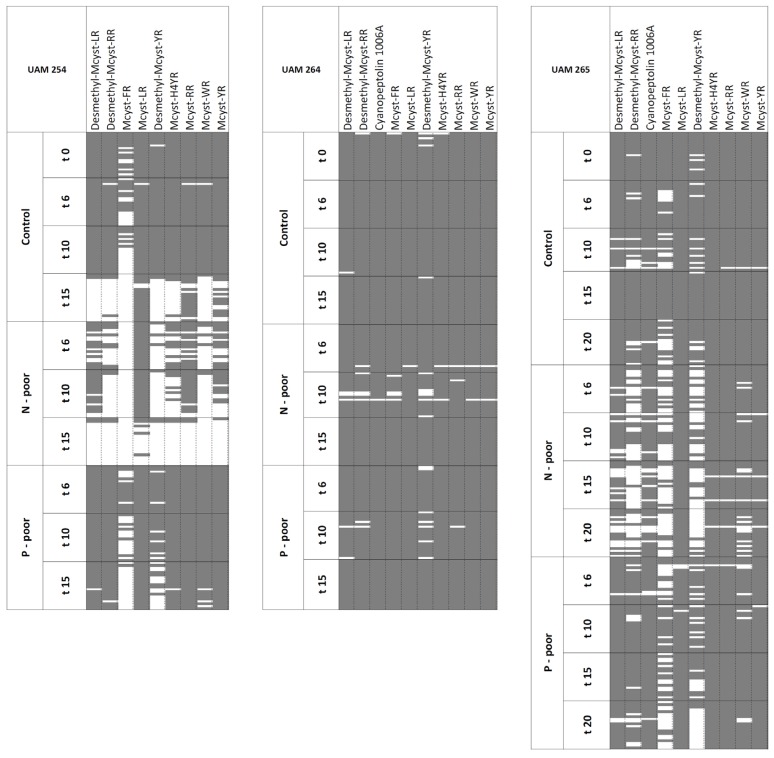
Presence/absence matrices of oligopeptides for strains UAM254, UAM264 and UAM265 under N- and P-poor treatments. Columns show previously described oligopeptides, whereas rows represent the analyzed samples, sorted by nutrient treatment applied and time of sampling (days). Filled/colored cells correspond to presence; blank cells stand for absence.

**Figure 2:**
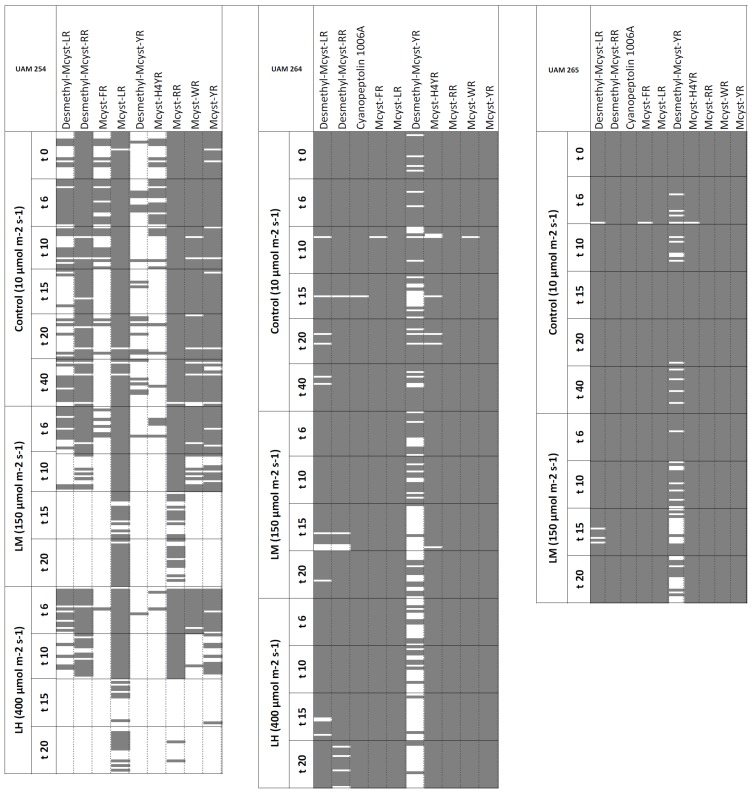
Presence/absence matrices of oligopeptides for strains UAM254, UAM264 and UAM265 under different light intensities. Columns show previously described oligopeptides, whereas rows represent the analyzed samples, sorted by light intensity treatment applied and time of sampling (days). Filled/colored cells correspond to presence; blank cells stand for absence.

A total of 1401 samples (colonies or groups of free cells) were analyzed by MALDI-TOF MS for their qualitative oligopeptide composition, generating a pool of more than 15,500 mass signals corresponding to, among others, 10 previously described oligopeptides [18]. Obtained data were used to construct an oligopeptide presence/absence matrix for each strain and condition (Figure 1, Figure 2). The selected strains produced mass spectra containing a wide range of putative MCs congeners and one cyanopeptolin variant (Table 1). Strain UAM254 showed a peptide composition consisting of a range of nine MC-variants. Strain UAM264 and UAM265 provided identical peptide patterns, with nine MC variants and one cyanopeptolin (cyanopeptolin 1006A, 1007.5 Da). The most intense signals corresponded to MC-LR, MC-RR and MC-YR, independently of the analyzed strain.

### 2.2. Growth Rates

We employed reductions in growth rates *versus* controls as a proxy of the fitness of the cultures under assayed conditions and, thereby, evaluate the effectiveness of treatments. N-poor treatment caused significant decreases in growth rates in two of the three analyzed strains, while P-poor only caused significant reductions in the growth rates of one strain (Table 2). UAM254 showed similar growth rates under control and P-poor treatments, significantly higher than those observed in the N-poor culture. Strain UAM264 did not show differences in growth rates between control and both N- and P-poor conditions. Nutrient treatments also induced reductions in growth rates in strain UAM265. In the light intensity experiment, non-colonial strain UAM254 exhibited under medium light (LM) and high light (LH) treatments significantly lower growth rates than control (low light; LL). Strain UAM264 also showed lower growth rates when compared to control, although differences were only statistically significant under LM treatment. Strain UAM265 did not present positive growth rates under high light conditions (LH) and started to decline a few days after inoculation. Although some colonies of this culture were analyzed, they presented poor pigmentation and produced only mass spectra with extremely low signals and were, hence, excluded from the analysis. Cultures under LM treatment presented significantly lower growth rates than under low light conditions (LL).

**Table 1:** Detected oligopeptides by MALDI-TOF MS for each analyzed strain and their respective protonated *m*/*z* ratio.

Peptide Name	Protonated *m*/*z* [M + H^+^] (Da)	UAM254	UAM264	UAM265
Desmethyl-Mcyst-LR	981.5	x	x	x
Mcyst-LR	995.6	x	x	x
Cyanopeptolin 1006A	1007.5		x	x
Desmethyl-Mcyst-RR	1024.6	x	x	x
Mcyst-FR	1029.5	x	x	x
Desmethyl-Mcyst-YR	1031.5	x	x	x
Mcyst-RR	1038.6	x	x	x
Mcyst-YR	1045.5	x	x	x
Mcyst-H4YR	1049.6	x	x	x
Mcyst-WR	1068.6	x	x	x

**Table 2:** Growth rates and relative intensity threshold for each strain and treatment.

Strain		Growth rates (day^−1^)	Relative intensity threshold ^(a)^
Strain UAM254	NC	0.146	0.04
	NN	0.108 ^(^*^)^	0.34
	NP	0.181	0.04
	LL	0.135	0.08
	LM	0.060 ^(^*^)^	0.25
	LH	0.070 ^(^*^)^	0.25
Strain UAM264	NC	0.274	0 (stable)
	NN	0.205	0 (stable)
	NP	0.275	0 (stable)
	LL	0.206	0 (stable)
	LM	0.117 ^(^*^)^	0.03
	LH	0.163 ^(^*^)^	0.03
Strain UAM265	NC	0.383	0 (stable)
	NN	0.172 ^(^*^)^	0.07
	NP	0.179 ^(^*^)^	0.06
	LL	0.190	0 (stable)
	LM	0.103 ^(^*^)^	0.03
	LH	-----	-----

^(a)^ Relative intensity threshold refers to the highest mean relative intensity among peptides, which presented interreplicate detection frequencies (IDFs) below 60% (*i.e.*, unstable) NC: nutrient control; NN: N-poor treatment; NP: P-poor treatment; LL: low light (Control), 10 μmol photons m^−2^s^−1^; LM: medium light, 150 μmol photons m^−2^s^−1^; LH: high light, 400 μmol photons m^−2^s^−1^; ^(^*^)^ Value significantly different to respective control treatments (ANOVA Holm-Sidak test; *p* < 0.05).

### 2.3. Stability under N- and P-Reduction

N-poor conditions did not cause changes in the oligopeptide pattern in strain UAM264. However, in the remaining strains, ascending hierarchical classification (AHC) did reveal variations under N-poor conditions (UAM254 and UAM265). Differences in the peptide patterns under the P-poor treatment could only be detected in one strain (UAM265).

Until the last days before achieving the stationary phase, strain UAM254 provided, under both control and P-poor conditions, spectra containing mass signals corresponding to all the range of MCs, except for MC-FR, which could not always be detected (Figure 1). However, during the last days before achieving the stationary growth phase (*t* = 15 days), oligopeptide detection under control conditions (nutrient control; NC) was dramatically reduced to signals corresponding to MC-LR, MC-YR and MC-RR. Under N-poor conditions, spectra lacked many of the mass peaks (Figure 1), which corresponded to low intensity oligopeptides, while only signals that exhibited high mean relative intensities (>0.34; Table 3) were conserved. In fact, oligopeptide compositions recorded in the N-poor replicates were clearly separated in different clusters from those recorded in the control (except control at *t* = 15 days; Table S1A). AHC did not evidence significantly different oligopeptide profiles in P-poor cultures and control treatment (except control at *t* = 15 days), as most of the replicates were grouped in the same cluster. 

**Table 3:** Interreplicate detection frequencies (IDFs) and average relative intensities of individual peptides. Peptides are classified as stable or unstable, according to their observed IDFs. Peptides with IDF ≤ 60% are considered unstable.

	IDF						Average relative intensities							IDF						Average relative intensities			
**Strain UAM254**	Total	NC	NN	NP			Total	NC	NN	NP			**Strain UAM254**	Total	LL	LM	LH			Total	LL	LM	LH
MC-LR	**91%**	96%	75%	100%	STABLE		**1.00**	1.00	1.00	1.00			MC-RR	**79%**	99%	76%	61%	STABLE		**0.97**	1.00	0.89	0.98
MC-RR	**84%**	94%	60%	100%	STABLE		**0.63**	0.73	0.39	0.65			MC-LR	**87%**	97%	91%	70%	STABLE		**0.61**	0.29	0.87	0.91
MC-YR	**78%**	89%	**42%**	100%	**UNSTABLE**		**0.34**	0.39	0.24	0.31			MC-YR	**59%**	91%	**37%**	**34%**	**UNSTABLE**		**0.25**	0.24	0.23	0.28
MC-H4YR	**71%**	80%	**33%**	98%	**UNSTABLE**		**0.18**	0.20	0.17	0.16			Desmethyl-MC-RR	**61%**	93%	**33%**	**41%**	**UNSTABLE**		**0.24**	0.25	0.15	0.29
Desmethyl-MC-LR	**76%**	77%	**52%**	98%	**UNSTABLE**		**0.18**	0.15	0.31	0.13			MC-WR	**56%**	93%	**34%**	**24%**	**UNSTABLE**		**0.16**	0.17	0.15	0.14
Desmethyl-MC-RR	**66%**	77%	**20%**	98%	**UNSTABLE**		**0.14**	0.15	0.15	0.13			Desmethyl-MC-LR	**32%**	**47%**	**20%**	**23%**	**UNSTABLE**		**0.08**	0.05	0.08	0.15
MC-WR	**61%**	75%	**10%**	95%	**UNSTABLE**		**0.11**	0.13	0.08	0.10			Desmethyl-MC-YR	**8%**	**18%**	**1%**	**1%**	**UNSTABLE**		**0.06**	0.06	0.04	0.07
Desmethyl-MC-YR	**53%**	76%	**10%**	67%	**UNSTABLE**		**0.08**	0.09	0.11	0.06			MC-FR	**14%**	**28%**	**7%**	**1%**	**UNSTABLE**		**0.03**	0.03	0.03	0.04
MC-FR	**26%**	37%	**0%**	37%	**UNSTABLE**		**0.04**	0.04	ND	0.04			MC-H4YR	**13%**	**25%**	**5%**	**3%**	**UNSTABLE**		**0.03**	0.03	0.04	0.05
**Strain UAM264**	Total	NC	NN	NP			Total	NC	NN	NP			**Strain UAM264**	Total	LL	LM	LH			Total	LL	LM	LH
MC-LR	**99%**	100%	97%	100%	STABLE		**0.99**	0.97	0.99	1.00			MC-RR	**100%**	100%	100%	100%	STABLE		**0.80**	1.00	0.71	0.60
MC-RR	**98%**	100%	95%	98%	STABLE		**0.81**	0.85	0.79	0.77			MC-LR	**100%**	100%	100%	100%	STABLE		**0.79**	0.51	0.99	0.99
MC-YR	**99%**	100%	95%	100%	STABLE		**0.47**	0.53	0.39	0.47			Cyanopeptolin 1006A	**100%**	99%	100%	100%	STABLE		**0.33**	0.15	0.36	0.56
MC-H4YR	**98%**	99%	95%	100%	STABLE		**0.34**	0.32	0.26	0.43			MC-YR	**100%**	100%	100%	100%	STABLE		**0.33**	0.29	0.38	0.33
Cyanopeptolin 1006A	**99%**	100%	97%	100%	STABLE		**0.30**	0.26	0.37	0.30			MC-WR	**100%**	99%	100%	100%	STABLE		**0.20**	0.17	0.25	0.18
MC-WR	**99%**	100%	95%	100%	STABLE		**0.23**	0.27	0.19	0.22			MC-FR	**100%**	99%	100%	100%	STABLE		**0.16**	0.10	0.17	0.23
MC-FR	**97%**	99%	92%	100%	STABLE		**0.10**	0.10	0.09	0.11			MC-H4YR	**98%**	96%	99%	100%	STABLE		**0.12**	0.06	0.17	0.13
Desmethyl-MC-RR	**96%**	99%	90%	97%	STABLE		**0.09**	0.09	0.10	0.09			Desmethyl-MC-RR	**97%**	99%	95%	95%	STABLE		**0.11**	0.14	0.09	0.08
Desmethyl-MC-LR	**97%**	99%	93%	97%	STABLE		**0.09**	0.08	0.11	0.09			Desmethyl-MC-LR	**95%**	95%	94%	96%	STABLE		**0.06**	0.04	0.06	0.07
Desmethyl-MC-YR	**91%**	95%	88%	88%	STABLE		**0.05**	0.05	0.05	0.05			Desmethyl-MC-YR	**52%**	75%	**47%**	**23%**	**UNSTABLE**		**0.03**	0.03	0.03	0.04
**Strain UAM265**	Total	NC	NN	NP			Total	NC	NN	NP			**Strain UAM265**	Total	LL	LM	LH			Total	LL	LM	LH
MC-LR	**99%**	100%	100%	96%	STABLE		**1.00**	1.00	1.00	1.00			MC-RR	**100%**	100%	100%		STABLE		**0.88**	1.00	0.71	
MC-H4YR	**98%**	100%	96%	99%	STABLE		**0.39**	0.32	0.28	0.58			MC-LR	**100%**	100%	100%		STABLE		**0.74**	0.57	0.99	
MC-RR	**98%**	99%	96%	99%	STABLE		**0.33**	0.38	0.26	0.34			MC-YR	**100%**	100%	100%		STABLE		**0.42**	0.38	0.48	
MC-YR	**98%**	99%	95%	99%	STABLE		**0.33**	0.38	0.28	0.32			MC-WR	**100%**	100%	100%		STABLE		**0.23**	0.20	0.27	
Cyanopeptolin 1006A	**92%**	96%	82%	96%	STABLE		**0.17**	0.15	0.13	0.23			Cyanopeptolin 1006A	**100%**	100%	100%		STABLE		**0.23**	0.13	0.36	
MC-WR	**90%**	99%	78%	91%	STABLE		**0.14**	0.16	0.13	0.13			MC-H4YR	**99%**	99%	100%		STABLE		**0.15**	0.09	0.23	
Desmethyl-MC-LR	**88%**	97%	69%	96%	STABLE		**0.13**	0.12	0.09	0.17			Desmethyl-MC-RR	**100%**	100%	100%		STABLE		**0.12**	0.14	0.10	
Desmethyl-MC-RR	**67%**	86%	**27%**	84%	**UNSTABLE**		**0.07**	0.06	0.04	0.08			MC-FR	**99%**	99%	100%		STABLE		**0.10**	0.08	0.14	
Desmethyl-MC-YR	**58%**	85%	**26%**	**56%**	**UNSTABLE**		**0.06**	0.06	0.04	0.07			Desmethyl-MC-LR	**98%**	99%	96%		STABLE		**0.06**	0.04	0.08	
MC-FR	**53%**	74%	**29%**	**50%**	**UNSTABLE**		**0.05**	0.05	0.04	0.05			Desmethyl-MC-YR	**77%**	88%	**60%**		**UNSTABLE**		**0.03**	0.03	0.05	

Strain UAM264 presented a conserved oligopeptide pattern under all treatments (Figure 1), as confirmed by AHC, which did not show separation between oligopeptide fingerprints in any of the nutrient treatments, when compared to control conditions, having most of the samples consistently assigned to the same cluster (Table S1B).

Strain UAM265 showed differences between peptide patterns under control conditions and those obtained in both N- and P- poor treatments (Table S1C). Under P-poor conditions, interreplicate detection frequencies (IDFs) corresponding to oligopeptides with the lowest relative intensity signals (Desmethyl-MC-YR and MC-FR) falling below 60%, leading to instability of the peptide fingerprint. N-poor treatment showed mass peaks corresponding to Desmethyl-MC-YR, MC-FR and also Desmethyl-MC-RR as non-stable signals, with IDFs below 30% (Table 3).

### 2.4. Stability under High Light Intensities

Increased light irradiation (both LM and LH) caused variations in the peptide compositions in all strains. Strain UAM254 provided spectra with incomplete oligopeptide fingerprints (Figure 2), even under control conditions (10 μmol m^−2^s^−1^), lacking microcystins that presented average low relative intensity signals (≤0.08; Table 3). Peptide compositions of all light treatments were similar during the first days: AHC grouped replicates of LL and both higher light intensities in the same cluster for the initial days of growth (*t* = 6 days). However, in peptide patterns obtained in later days (*t* = 15, 20 days), some peptides were missing, and replicates were therefore grouped in a different cluster by AHC, thereby evidencing variations in the oligopeptidic fingerprint (Table S2A). Under increased light conditions, mass signals corresponding to unstable oligopeptides showed always average relative intensities below 0.25 (Table 3).

Oligopeptide compositions of strains UAM264 and UAM265 were consistent along treatments (Figure 2), with the exception of signal corresponding to Desmethyl-MC-YR, which showed the lowest relative intensities and could only be detected with IDFs below 60% under higher light intensities (Table 3). Based on its absence, AHC evidenced differences in peptide fingerprints under LM (both strains) and LH treatments (UAM264), when respectively compared to LL treatment (Table S2B,C).

### 2.5. Extent of the Peptide Fingerprint Stability

In general, a significant reduction in growth rates caused by treatments was followed by a gradual disappearance of microcystins mass signals with increasing relative intensities from oligopeptide profiles. The extent of the instability in the oligopeptidic fingerprint could hence be expressed as a relative intensity threshold, above which oligopeptides could be consistently detected. This threshold served as a metric of the extent of the instability, as well as a comprehensive delimitation between stable and unstable oligopeptides, according to the IDF criteria set. Strain UAM264 did not exhibit significant reductions in growth rates under nutrient limiting conditions and presented a stable oligopeptide fingerprint. In the light treatments though, where reduction in growth rates was significant (Table 2), differences were caused by Desmethyl-MC-YR, which was detected with the lowest mean relative intensity (0.03). Instability in the peptide fingerprint of strain UAM265 was caused by the absence of more peptides, whose maximal mean relative intensity (*i.e.*, relative intensity threshold) ascended to 0.07 (Table 3). Non-colonial strain UAM254, showing the lowest growth rates, provided incomplete peptide compositions, even under control conditions, presenting even higher relative intensity thresholds (0.34 and 0.24 in nutrient and light experiments, respectively; Table 3).

## 3. Discussion

MALDI-TOF MS has been pointed out as a useful and fast screening technique for the detection of cyanobacterial peptides, toxins and other bioactive compounds [8,19,20]. Recent studies dealt with the description of oligopeptide-based subspecific units or chemotypes, their ecology, dynamics and diversity [9,10,14,21]. However, this approach was based on the assumption that qualitative cyanobacterial oligopeptide composition was a stable feature, unaffected by abiotic conditions. Although there is abundant bibliography about the factors affecting synthesis of single oligopeptides [14,15,16] and cyanobacterial cultures have been observed to produce oligopeptides cyanopeptolin and microcystin for years under constant laboratory conditions [22,23], to our knowledge, no specific studies have related the observed differences in oligopeptide production under changing conditions in the context of the definition of chemotypes. Evaluating the consistency and conservation of oligopeptide fingerprints under varying environmental conditions, as well as understanding to what extent these are reliable in the frame of chemotype delimitation, is central for the correct interpretation of the dynamics of chemotypical subpopulations under field conditions.

In our study, the different extent of the reduction in growth rates under different treatments evidences that strains show disparate susceptibility to nutrient deficiency. Intraspecific variations in growth rates under identical conditions have previously been described [24]. While strain UAM264 grew with similar rates under all nutrient treatments during the studied period, UAM265 suffered both N- and P-deficiency (Table 2). Strain UAM254 exhibited overall lower growth rates, which may be attributed to its non-colonial morphology [25]. All in all, our observations evidence that strains may show different growth optima, even when belonging to the same chemotype (UAM264 and UAM265). In line with the varying susceptibility to changing nutrient and light conditions, we also found a different response among strains in terms of stability of their oligopeptide profiles. While peptide composition changed in strains UAM254 and UAM265 when nutrient and/or light conditions were altered, strain UAM264 conserved its original peptide pattern. Interestingly, variations in microcystin compositions were only observable when treatments caused significant reductions in growth rates. Strains unaffected by treatments (*i.e*., no statistically significant reductions in growth rates) provided highly conserved peptidic profiles. Indeed, missing oligopeptides always provided the lowest average relative intensities in the mass spectra (Table 3).

The gradual disappearance of low intensity producing peptides enabled the use of a relative intensity threshold as an indicator of the extent of the oligopeptide fingerprint stability. Microcystins with average relative intensities below the relative intensity threshold (RIT) value could not be consistently detected. In other words, RITs can potentially be used as a threshold to discern between stable and unstable peptides. Together with the number of unstable peptides, RIT values were also higher when greater reductions in growth rates were observed (Table 2), denoting that the fitness of the culture, and the degree of conservation of microcystin signatures are closely linked.

In light of these observations, we hypothesize that physiological effects caused by intense light irradiation and/or nutrient limitation, especially N-deficiency, causes an overall reduction in microcystins cellular concentrations, leading to alterations in the oligopeptide profiles employed for chemotype delimitation. Under high light conditions, free microcystin concentration in the cell may be reduced as a result of covalent binding of microcystin to the cysteine residues of proteins involved in photosynthesis, as recently demonstrated by Zilliges *et al*. [26]. This binding was observed to be dramatically enhanced under high light conditions, suggesting that microcystins may play a role as a protein-modulating metabolite under oxidative stress conditions. Alternatively, a decrease in the NPRS/PKS enzyme complex activity as a result of physiological stress would explain an overall reduction in MC synthesis: The coexistence of multiple MC variants partially results from the NPRS/PKS multi-enzyme complex substrate inspecificity in the incorporation of amino acids in positions 2 and 4 [27]. A reduction in the activity of the NRPS/PKS enzyme complex would therefore reduce the synthesis of all coexisting MC congeners homogeneously, causing minority MC variants (*i.e*., those providing the lowest intensity signals) to fall below detection limits. Reduced NPRS activities under physiological stress are consistent with previous findings demonstrating that maximal oligopeptide concentrations are found under optimal growth conditions [15,16] and that MC production is directly and linearly correlated with growth rates [12]. A further plausible explanation refers to reductions in the availability of amino acids involved in oligopeptide synthesis as a result of N-limitation. Similarly, increased photosynthesis under high light irradiation would have an equivalent effect, raising the C/N cellular ratio and ultimately leading to N-deficiency. Nitrogen availability modulates the cellular amino acid composition, reducing nitrogen-rich amino acids, such as arginine, under limiting conditions [28]. All detected microcystin variants in our study contained one arginine in their structure, so that overall microcystin production would be homogenously reduced. Exceptionally, variants MC-RR and Desmethyl-MC-RR contain arginine in both positions 2 and 4. Interestingly, we observed reductions in the relative intensity of signals corresponding to MC-RR in favor of MC-LR (the co-dominant variant in the mass spectra) under both high light and N-poor conditions (Table 3). Reductions in MC-RR production among other variants under N-limiting conditions and high light intensities have been reported previously [28,29], evidencing that its synthesis is closely related to arginine availability. Desmethyl-MC-RR is also probably affected in this way, but changes in signals with initially low absolute intensities remain negligible in terms of relative intensity. 

Previous studies reported that MALDI-TOF MS shows preferential ionization of peptides containing arginine in their structure [30]. One could therefore concede that MALDI-TOF results may not represent the complete cellular oligopeptide composition, having non-arginine-containing peptides as underestimated signals close to or even below detection limits. As all MC variants detected in our study contained arginine, the presence of further oligopeptides cannot be discarded. However, variations in a peptide pattern with respect to controls (presence/absence or changes in relative intensities) have to be regarded as actual variations in the oligopeptide net production that transcend the mere alteration of the peptide fingerprint, raising important implications regarding the definition of the chemotype itself. For instance, UAM264 and UAM265 presented identical peptide compositions under control conditions and have therefore to be considered the same chemotype. However, in light of the observed variations in the general oligopeptide pattern under N and P limitation, the dilemma is evident: the same chemotype under nutrient rich conditions or two distinct chemotypes under limiting conditions?

The oligopeptide profiles of the selected strains in our study only represent a portion of the complete array of oligopeptides typically used for chemotype delimitation, and the observed fingerprint instability was caused by the loss of microcystin signatures. Although most oligopeptides are produced via non-ribosomal biosynthetic pathways, a generalization of our findings to other oligopeptide classes has to be done with caution. Further investigations should, hence, focus on addressing a wider range of bioactive compounds to assess whether our findings are applicable to other oligopeptide classes. Still, the distortion of oligopeptidic fingerprints, although consisting solely of microcystins, constitutes an unreported issue in the context of chemotypes definition. Assessing the instability of oligopeptide compositions is crucial for an unbiased interpretation of the dynamics of chemotypical subpopulations in natural systems. However, homogeneous criteria for chemotype delimitation are currently not available. In fact, studies dealing with the dynamics of chemotypical subpopulations show huge differences in terms of chemotype diversity. For instance, Rohrlack *et al*. [10] identified four *Planktothrix* chemotypes in Lake Steinsfjorden throughout a period of 33 years. In contrast, Welker *et al*. [9] found 37 different *Microcystis* chemotypes in Brno reservoir in a study period of five months. Although these studies focus on different genera and may not be comparable, chemotype delimitation was performed in each case using different methodologies. On the one hand, the inclusion of strains with similar, but not identical oligopeptide patterns in the same chemotype (Lake Steinsfjorden) lead to the definition of a low number of subpopulations. On the other hand, data on Lake Brno was analyzed on the basis that changes in individual oligopeptides implied a different chemotypical unit. Our study shows that such variations may occur within clonal subpopulations, suggesting that the number of chemotypes in Lake Brno could partially be overestimated. Sudden changes in abiotic conditions (e.g., nutrient inputs), together with the differences in physiological conditions among individuals within the same chemotype, may trigger changes in the recorded oligopeptide profiles, which may be misinterpreted as shifts in the chemotypical subpopulations, while the clonal composition of the population actually remains unchanged. Therefore, we believe that chemotypical delimitation needs to be done on the basis of the presence of high intensity oligopeptides, as the detection of low intensity producing microcystins has been shown to be highly dependent on the physiological condition of the subpopulation and is, therefore, not always possible. Thus, setting a relative intensity threshold to identify reliable (*i.e*., stable) oligopeptides for chemotype definition seems a plausible option to circumvent this problem. However, a definition of a relative intensity threshold to generally discern between “stable” and “potentially unstable” oligopeptides is still needed, given the differences in susceptibility to stressing conditions among strains and the variability in detection limits in the MALDI-TOF technique from laboratory to laboratory. Further research should, hence, focus on describing the relationship between the degree of instability in the oligopeptidic patterns (e.g., via RIT) and specific physiological indicators, which can normalize the observed intraspecific variations in growth optima. Still, our findings provide unreported evidence for variability in the detection of microcystin signatures, which may lead to a biased delimitation of oligopeptide-based chemotypes. While restricted to microcystins, such variability affects oligopeptide signatures as a whole and needs to be addressed to allow the definition of consistent chemotypical subpopulations that can be unequivocally identified under the complete range of natural conditions.

## 4. Experimental Section

### 4.1. *Microcystis aeruginosa* Strains

Three strains of *M. aeruginosa* were used, namely UAM254 (non-colonial morphotype), UAM264 and UAM265 (colonial morphotypes), which were isolated between 1998 and 2000 from Santillana Reservoir, a eutrophic and typically monomictic water body located in Madrid, central Spain. Limnological features of the reservoir are described elsewhere [31]. After isolation, the strains were maintained as clonal cultures in a BG11 medium [32] under constant conditions at 28 °C and 10 μmol photons m^−2^ s^−1^ illumination provided by continuous cool white fluorescent light.

### 4.2. Experimental Setup

Nutrient treatments were cultured under continuous light irradiance of 70 μmol photons m^−2^s^−1^. All experiments were conducted after 2 weeks acclimation period to assayed conditions. Nutrient control (NC) conditions used the original BG11 medium ([P] = 175 µM; [N] = 2 mM). N-poor (NN) treatment consisted in the use of a modified BG11 medium containing only 10% of the original NaNO_3_ concentration ([N] = 200 µM), while P-poor treatment (NP) consisted in a modified BG11 medium containing only 10% of the original K_2_HPO_4_ concentration ([P] = 17.5 µM). Glassware containing P-poor medium was washed previously with HCl (10%) to eliminate P traces. Light experiments were carried out using the original BG11 medium under different continuous light intensities: low light (LL) conditions consisted in a 10 μmol photons m^−2^s^−1^ irradiance and were used as control for the comparison of oligopeptide compositions, while medium (LM) and high light (LH) conditions provided 150 and 400 μmol photons m^−2^s^−1^, respectively. Sterile glassware with treatment-specific nutrient medium was inoculated with 1 mL aliquots of the original cultures in the exponential phase and placed under the appropriate light conditions. The resulting cultures were maintained under the same temperature, and their growth was monitored by spectrophotometric readings at 750 nm. Culture growth rates were calculated for each treatment during the exponential phase [μ = ln(Nt/No)/Δ*t*]. 

### 4.3. MALDI-TOF MS Analysis

During the active growth phase and until the stationary growth phase was achieved, individual colonies or free cells were collected for the determination of their oligopeptide compositions through MALDI-TOF MS. Twenty replicates for each strain-treatment-time combination were analyzed. Colony biovolume (for colonial strains) and optical density (for unicellular cultures) were correlated to cell numbers for each strain (data not shown), were used to indirectly ensure sufficient biomass in the samples (>6000 cells) and, thereby, obtain unambiguous mass spectra. Single colonies of at least 0.03 mm^3^ or 5 μL of free cell suspension were collected with disposable glass capillaries and placed in 0.2 mL Eppendorf tubes, which were allowed to dry at room temperature during 2 h. Ten microliters of an acetonitrile, ethanol and water (1:1:1) extractant solution, acidified with 0.03% (*v*/*v*) trifluoroacetic acid (TFA), were then added to each sample. Samples were introduced into liquid nitrogen for 2 h to induce cell lysis and stored at −80 °C until analyzed. For MS analysis, 0.5 μL of individual samples analyte solution were placed onto the matrix spots of a Prespotted AnchorChip (PAC 284/96 HCCA, Bruker Daltonics, Bremen, Germany), which contain α-cyano-4-hydroxycinnamic acid (HCCA) as co-crystallizing matrix. Spots were allowed to dry at room temperature and were washed with ammonium phosphate (10 mM) for 5 s before MALDI-TOF analysis. Mass spectrometric analysis was performed on a Bruker Reflex MALDI mass spectrometer equipped with a time of flight (TOF) mass analyzer on positive ion detection mode and reflector mode. Acceleration voltage was set to 25 kV and mirror voltage to 26.3 kV. Mass spectra were accumulated from 1000 laser pulses scanning the entire sample spot for a mass range of 500–2000 Da.

### 4.4. Data Processing

The resulting mass spectra were processed using Bruker FlexAnalysis software (version 3.0). The SNAP algorithm (Bruker Daltonics, Bremen, Germany) was used for mass determination using a signal to noise threshold of 6. Putative oligopeptides were identified according to their mass/charge ratio, compared with previously described oligopeptides [18]. The occurrence of Na and K adducts in the ionization was also examined, and their assignment was corrected to the appropriate protonated oligopeptide. Mass signals that could not be assigned to any previously described oligopeptide were excluded from the analysis. Relative intensities were calculated based on the absolute signal intensity of the highest identified oligopeptide peak in the mass spectrum. Obtained data were used to construct a matrix for each strain, illustrating the presence or absence of oligopeptides along the different treatments and sampling times. To evaluate the differences between the oligopeptide fingerprints recorded under different treatments, an ascending hierarchical clustering (AHC) was conducted: for each strain, multiple datasets consisting of each treatment and its respective control were constructed. The resulting individual datasets were examined by AHC, which analyzed dissimilarities using Euclidean distances and applying Ward’s agglomeration method. The number of clusters was predefined (*k* = 2), so that data variability within the dataset was separated into two clusters. The resulting clusters contained samples with similar (or identical) peptide compositions.

To evaluate whether applied treatments caused changes in the peptide composition compared to controls, the percentage of treatment and control replicates assigned to each cluster was examined. When treatments induced variations in the peptide fingerprint, control replicates were majorly and selectively concentrated in cluster A, while most of the treatment replicates were grouped in cluster B. Conversely, when assayed conditions did not cause changes in the peptide composition, non-selective assignment occurred, having samples corresponding to both control and treatment majorly assigned to the same cluster.

Given that chemotypical subpopulations are delimited by their respective peptide compositions, which are usually defined by the presence of individual oligopeptides, the gain or loss of one or more peptides would imply a change in the chemotype-specific fingerprint. Conserved oligopeptide patterns were, hence, considered those which conserved their complete oligopeptide combination regardless of treatments and time. Therefore, stability was analyzed as the consistency in detection of individual peptides. Detection frequencies were expressed as a percentage of positive detections among same-treatment replicates (termed onwards IDF, interreplicate detection frequency) and aim to provide a metric of the variations in detection of individual peptides between replicates. Oligopeptides presenting IDFs below 60% were regarded as unstable signals and consequently considered to account for changes in the oligopeptide fingerprint. This value aimed to provide a conservative limit to discern between stable and unstable peptides. To express the extent of the fingerprint instability, a relative intensity threshold (RIT) was calculated, which represents the maximal mean relative intensity among unstable oligopeptides (*i.e*., those showing IDFs below 60%). RIT values represent the delimitation, in terms of mass spectral relative intensity, between stable and unstable peptides for each treatment and strain.

## 5. Conclusions

The stability of oligopeptide fingerprints used for the definition of cyanobacterial chemotypes has not been assessed. Our results evidence that oligopeptide fingerprints may be subject to distortion under suboptimal physiological conditions, consisting in the gradual loss of low intensity signals corresponding to minor microcystin variants. Changes in the oligopeptide patterns may be misinterpreted as shifts in the composition of chemotypes and may lead to overestimation of their diversity in natural populations. Therefore, criteria for the definition of oligopeptide chemotypes should be based on the detection of high intensity producing peptides, which can be consistently detected over the complete range of environmental conditions, allowing an accurate analysis of the dynamics of chemotypes in natural ecosystems. 

## Supplementary Files

Supplementary File 1 Supplementary Information (PDF, 90 KB)
