# Supplementary material for: Limited Stability of Microcystins in Oligopeptide Compositions of Microcystis aeruginosa (Cyanobacteria): Implications in the Definition of Chemotypes
Source: Toxins (Basel). 2013 Jun 6;5(6):1089–104. doi: 10.3390/toxins5061089 (PMC3717771; doi:10.3390/toxins5061089)
Supplement: Supplementary File 1 — Supplementary Information (PDF, 90 KB) [file toxins-05-01089-s001.pdf]

Supplementary Information

Table S1. Results of ascending hierarchical clustering for nutrient treatments. N-deficient (NN) and P-deficient (NP) oligopeptide compositions were, respectively, compared to control conditions (NC). The assignment to each cluster is expressed as a percentage of treatment replicates. Different treatments are codified according to strain name, treatment applied and day of sampling (*i.e.*, 265 NC t10 corresponds to UAM265, control conditions, day 10). *n* stands for number of replicates analyzed in each case.

**Strain UAM254**								**A**
NN *vs*. NC					NP *vs*. NC			
Treatment	*n*	Cluster A	Cluster B		Treatment	*n*	Cluster A	Cluster B
254 N t0	19	95%	5%		254 N t0	19	100%	0%
254 NC t6	20	95%	5%		254 NC t6	20	100%	0%
254 NC t10	20	100%	0%		254 NC t10	20	100%	0%
254 NC t15	20	5%	95%		254 NC t15	20	20%	80%
**254 NC**	**79**	**73%**	27%		**254 NC**	**79**	**80%**	20%
254 NN t6	20	15%	85%		254 NP t6	20	100%	0%
254 NN t10	20	5%	95%		254 NP t10	20	100%	0%
254 NN t15	20	0%	100%		254 NP t15	20	100%	0%
**254 NN**	**60**	7%	**93%**		**254 NP**	**60**	**100%**	0%
**Strain UAM264**								**B**
NN *vs*. NC					NP *vs*. NC			
Treatment	*n*	Cluster A	Cluster B		Treatment	*n*	Cluster A	Cluster B
264 N t0	20	95%	5%		264 N t0	20	85%	15%
264 NC t6	20	100%	0%		264 NC t6	20	100%	0%
264 NC t10	20	100%	0%		264 NC t10	20	95%	5%
264 NC t15	20	100%	0%		264 NC t15	20	95%	5%
**264 NC**	**80**	**99%**	1%		**264 NC**	**80**	**94%**	6%
264 NN t6	20	95%	5%		264 NP t6	19	95%	5%
264 NN t10	19	84%	16%		264 NP t10	20	75%	25%
264 NN t15	20	100%	0%		264 NP t15	21	100%	0%
**264 NN**	**59**	**93%**	7%		**264 NP**	**60**	**90%**	10%
**Strain UAM265**								**C**
NN *vs*. NC					NP *vs*. NC			
Treatment	*n*	Cluster A	Cluster B		Treatment	*n*	Cluster A	Cluster B
265 N t0	20	85%	15%		265 N t0	20	85%	15%
265 NC t6	20	65%	35%		265 NC t6	20	65%	35%
265 NC t10	18	50%	50%		265 NC t10	18	50%	50%
265 NC t15	20	95%	5%		265 NC t15	20	95%	5%
265 NC t20	19	42%	58%		265 NC t20	19	42%	58%
**265 NC**	**97**	**68%**	32%		**265 NC**	**97**	**68%**	32%
265 NN t6	20	25%	75%		265 NP t6	20	45%	55%
265 NN t10	20	25%	75%		265 NP t10	20	50%	50%
265 NN t15	20	15%	85%		265 NP t15	20	40%	60%
265 NN t20	20	10%	90%		265 NP t20	20	5%	95%
**265 NN**	**80**	19%	**81%**		**265 NP**	**80**	35%	**65%**

Table S2. Results of ascending hierarchical clustering for light intensity treatments. One-hundred fifty micro-mole photons m-2 s-1 (LM) and 400 µmol photons m-2 s-1 (LS) oligopeptide compositions were, respectively, compared to low light (LL). The assignment to each cluster is expressed as a percentage of treatment replicates. Different treatments are codified according to strain name, treatment applied and day of sampling (*i.e.*, 265 LL t10 corresponds to UAM265, low light conditions (control), day 10). *n* stands for number of replicates analyzed in each case.

**Strain UAM254**							**A**
LM *vs*. LL				LH *vs*. LL			
Treatment	*n*	Cluster A	Cluster B	Treatment	*n*	Cluster A	Cluster B
254 L t0	20	100%	0%	254 L t0	20	100%	0%
254 LL t6	20	100%	0%	254 LL t6	20	100%	0%
254 LL t10	18	89%	11%	254 LL t10	18	94%	6%
254 LL t15	19	95%	5%	254 LL t15	19	100%	0%
254 LL t20	19	84%	16%	254 LL t20	19	95%	5%
254 LL t40	20	80%	20%	254 LL t40	20	85%	15%
**254 LL**	**116**	**91%**	9%	**254 LL**	**116**	**96%**	4%
254 LM t6	20	95%	5%	254 LH t6	20	95%	5%
254 LM t10	16	38%	63%	254 LH t10	19	79%	21%
254 LM t15	20	0%	100%	254 LH t15	20	0%	100%
254 LM t20	20	0%	100%	254 LH t20	20	0%	100%
**254 LM**	**76**	33%	**67%**	**254 LH**	**79**	**43%**	**57%**
**Strain UAM264**							**B**
LM *vs*. LL				LH *vs*. LL			
Treatment	*n*	Cluster A	Cluster B	Treatment	*n*	Cluster A	Cluster B
264 L t0	19	79%	21%	264 L t0	19	79%	21%
264 LL t6	20	90%	10%	264 LL t6	20	90%	10%
264 LL t10	20	75%	25%	264 LL t10	20	75%	25%
264 LL t15	19	37%	63%	264 LL t15	19	37%	63%
264 LL t20	19	84%	16%	264 LL t20	19	84%	16%
264 LL t40	20	80%	20%	264 LL t40	20	80%	20%
**264 LL**	**117**	**73%**	27%	**264 LL**	**117**	**75%**	25%
264 LM t6	19	63%	37%	264 LH t6	**20**	30%	70%
264 LM t10	20	70%	30%	264 LH t10	**20**	35%	65%
264 LM t15	20	10%	90%	264 LH t15	**20**	15%	85%
264 LM t20	20	45%	55%	264 LH t20	**20**	15%	85%
**264 LM**	**79**	**47%**	**53%**	**264 LH**	**80**	24%	**76%**

Table S2. *Cont.*

**Strain UAM265**			**C**
LM *vs.* LL			
Treatment	*n*	Cluster A	Cluster B
265 L t0	19	89%	11%
265 LL t6	20	60%	40%
265 LL t10	20	25%	75%
265 LL t15	20	80%	20%
265 LL t20	20	95%	5%
265 LL t40	20	35%	65%
**265 LL**	**119**	**65%**	35%
265 LM t6	20	70%	30%
265 LM t10	20	15%	85%
265 LM t15	20	0%	100%
265 LM t20	20	30%	70%
**265 LM**	**80**	29%	**71%**

© 2013 by the authors; licensee MDPI, Basel, Switzerland. This article is an open access article distributed under the terms and conditions of the Creative Commons Attribution license (http://creativecommons.org/licenses/by/3.0/).
